# A Review of Readout Circuit Schemes Using Silicon Nanowire Ion-Sensitive Field-Effect Transistors for pH-Sensing Applications

**DOI:** 10.3390/bios15040206

**Published:** 2025-03-22

**Authors:** Jungho Joo, Hyunsun Mo, Seungguk Kim, Seonho Shin, Ickhyun Song, Dae Hwan Kim

**Affiliations:** 1 Department of Intelligent Semiconductor and Display Engineering, Kookmin University, Seoul 02707, Republic of Korea; wnwjdgh0501@kookmin.ac.kr; 2 School of Electrical Engineering, Kookmin University, Seoul 02707, Republic of Korea; tyche@kookmin.ac.kr (H.M.); tmdrnr1220@daum.net (S.K.); 3 Department of Artificial Intelligence Semiconductor Engineering, Hanyang University, Seoul 04763, Republic of Korea; a5731020@hanyang.ac.kr; 4 Department of Electronic Engineering, Hanyang University, Seoul 04763, Republic of Korea

**Keywords:** ion-sensitive field-effect transistor (ISFET), N-type/P-type circuit, pH sensor, readout scheme, silicon nanowire (SiNW)

## Abstract

This paper reviews various design approaches for sensing schemes that utilize silicon nanowire (SiNW) ion-sensitive field-effect transistors (ISFETs) for pH-sensing applications. SiNW ISFETs offer advantageous characteristics, including a high surface-to-volume ratio, fast response time, and suitability for integration with complementary metal oxide semiconductor (CMOS) technology. This review focuses on SiNW ISFET-based biosensors in three key aspects: (1) major fabrication processes and device structures; (2) theoretical analysis of key performance parameters in readout circuits such as sensitivity, linearity, noise immunity, and output range in different system configurations; and (3) an overview of existing readout circuits with quantitative evaluations of N-type and P-type current-mirror-based circuits, highlighting their strengths and limitations. Finally, this paper proposes a modified N-type readout scheme integrating an operational amplifier with a negative feedback network to overcome the low sensitivity of conventional N-type circuits. This design enhances gain control, linearity, and noise immunity while maintaining stability. These advancements are expected to contribute to the advancement of the current state-of-the-art SiNW ISFET-based readout circuits.

## 1. Introduction

In modern drug development and disease treatment, early diagnosis and testing are critical as they assess a patient’s current status and provide key information for the next stages. Typically, early diagnosis has been conducted through various biomolecule-sensing methods, including electrochemical detection [1,2], optical detection [3,4], and mass spectrometry [5,6]. Electrochemical detection measures electrical changes resulting from chemical reactions between target biomolecules and functionalized elements (primarily enzymes), using their specific binding and catalytic properties to detect and quantify biomolecules [7]. Optical detection uses the ability of target molecules to absorb or emit light. Mass spectrometry is employed for the detection of target molecules, directly measuring their mass. However, these conventional methods are often limited by lengthy detection times, high costs, complex equipment, and the need for skilled operators and specialized laboratory environments, constraining their practicality in terms of accessibility, maintenance, and scalability.

In this regard, semiconductor-based biosensor technologies have received significant attention these days [8,9,10,11]. Semiconductor biochips have several advantages, such as the ability to perform point-of-care diagnostics without spatial or temporal constraints. In addition, these biochips can be miniaturized, making them more practical and cost-efficient for large-scale manufacturing environments [9,12,13]. Moreover, these technologies leverage the electrical properties of semiconductor devices to detect biological changes, enabling applications in diverse fields such as medical diagnostics and chemical analysis [14,15]. For example, field-effect transistor (FET)-based biosensors utilizing complementary metal oxide semiconductor (CMOS) technology have been used due to their high sensitivity and rapid response in detecting SARS-CoV-2 spike proteins, enabling effective pandemic-related diagnostics [16].

The development of semiconductor biochips involves the utilization of various types of transistors, with CMOS technology being one of the most widely used platforms. CMOS-based biochips integrate transistors and physical sensing materials on a single chip, reducing costs while enabling multi-functionality in a compact form factor. In general, CMOS biosensors offer advantages such as low power consumption, multi-modality sensing, short response time, and label-free detection [17,18,19,20,21,22,23,24,25,26]. In the literature, graphene field-effect transistor (GFET) technology leverages the unique properties of the two-dimensional carbon material graphene [27,28,29,30,31,32,33]. GFETs enable fast sensing capabilities due to their high electrical conductivity, and they enhance a signal-to-noise ratio (SNR) due to low electrical noise characteristics [34]. Next, ion-sensitive FETs (ISFETs), first proposed by Bergveld in 1970 [35], have been widely used for biomolecule detection. Unlike traditional three-electrode setups, ISFETs require only a single reference electrode, simplifying instrumentation. In addition, as robust solid-state sensors, ISFETs demonstrate high resistance to acidic and alkaline environments as well as physical damage [36,37].

Among various semiconductor technologies, nanoscale ISFETs in the form of a silicon nanowire (SiNW) have attracted significant attention for overcoming the physical limitations inherent in sensors fabricated with planar semiconductor technologies [38,39,40,41,42]. Their nanoscale structure enhances sensitivity, allowing for the detection of ultralow concentrations of biomolecules [43,44], while their reduced footprint makes them well-suited for high-density sensor arrays [42,45]. Additionally, SiNW ISFETs offer several advantages such as a high surface-to-volume ratio [46], real-time monitoring [8], and label-free detection capabilities like CMOS sensors [8,43,47,48,49]. These properties have been applied in detecting pH concentrations [50,51,52,53], nucleic acids [48,54,55,56,57,58,59,60], proteins [47,61,62,63,64,65,66,67,68], cells [69,70,71], protein–DNA interactions [20,25,72,73,74,75], and single-virus detection [76]. In addition, SiNW ISFETs are increasingly being integrated with artificial intelligence (AI), machine learning (ML), and internet-of-things (IoT) frameworks to enhance biosensor capabilities [77,78]. ML-optimized models have demonstrated higher precision and reliability than conventional physics-based models while maintaining robustness against environmental variations and noise, making them well-suited for real-time biosensor applications [79]. Moreover, SiNW ISFETs offer key advantages, including high reproducibility and compatibility with standard CMOS technology. These benefits have been demonstrated in biomolecular diagnostic systems by integrating top-down-fabricated SiNW ISFETs with CMOS circuits [80,81].

In the field of pH sensing, ISFET-based techniques fundamentally involve the changes in threshold voltage (V_TH_) due to ion concentration [82]. To enhance ISFET performance, many studies have focused on improving sensitivity, reducing noise, and mitigating environmental effects. To increase sensitivity, methods such as the extended gate have been proposed [83]. For noise suppression, studies have investigated aptamer-based functionalization, which selectively captures target molecules and prevents the adhesion of interfering substances, thereby enhancing the SNR [84,85]. In addition, differential ISFET configurations [86] and signal conditioning techniques [87] have been proposed to achieve temperature compensation. Research has also focused on electrode modifications using gold nanoparticles (AuNPs) to enhance potential signal strength [88,89]. A surface modification with aromatic diazonium salt membranes has been examined to precisely control the anchoring layer thickness, aligning it with the Debye length for optimized sensor performance [90,91]. These advancements contribute to enhancing biosensor accuracy and reliability.

**Figure 1:**
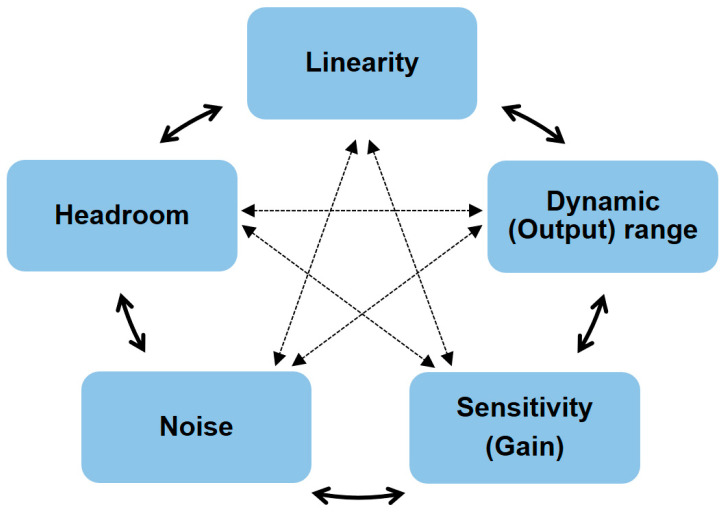
Trade-offs in semiconductor biochip design among linearity, dynamic (output) range, sensitivity (gain), noise, and headroom.

**Figure 2:**
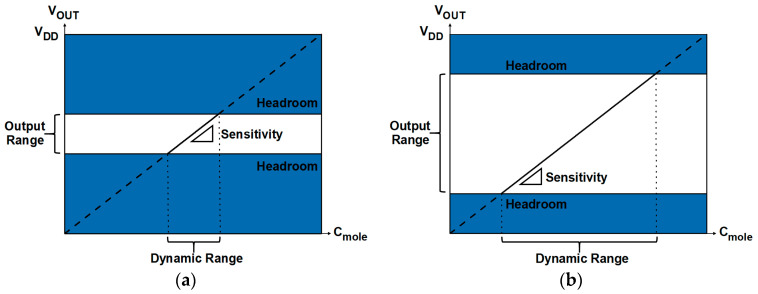
Relation between output and dynamic ranges with different headroom. Regions with solid lines denote available output ranges. (**a**) Narrower output and dynamic ranges due to small headroom; (**b**) wider output and dynamic ranges due to large headroom.

**Figure 3:**
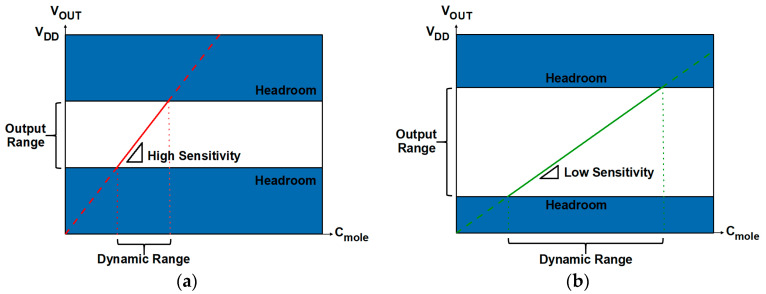
Trade-off between sensitivity and DR in biosensor design: (**a**) a narrower DR due to higher sensitivity; (**b**) a wider DR due to lower sensitivity. Regions with solid lines denote available output ranges.

The design of readout systems requires careful optimization of critical performance parameters, including dynamic range (DR), resolution, output range, sensitivity (gain), and linearity (Figure 1). In general, a wider DR is advantageous because DR represents the concentration range of target biomolecules (C_mole_) that can be sensed by readout circuits. The resolution refers to the smallest change in the input that can be reliably detected by the readout system regardless of DR; a higher resolution indicates an ability to distinguish finer variations [12]. The output range determines the maximum and minimum limits of the output signal that the circuit can generate, which directly affects DR. If the output range is too narrow, the DR may be limited, resulting in distortions or loss of information. A higher gain is typically preferred, but it potentially degrades linearity, indicating complex trade-offs that need to be considered. In readout circuit design, it is essential to manage the balance among these parameters. Figure 2 and Figure 3 illustrate the relationship between key properties of the system. It is shown that a voltage headroom affects the available output and DR (Figure 2), whereas a high sensitivity lowers a DR (Figure 3).

The existing review articles [92,93,94,95] have mainly focused on the general applications of ISFET sensors, particularly the detection of biomolecules such as DNA, proteins, and antibodies, and their commercialization potential. However, these reviews lack a critical analysis of recent circuit design technologies specific to SiNW ISFETs and their impact on sensor performance. For example, Cao et al. [92] discuss the applications of ISFET sensors in biomolecule detection and their commercialization prospects but provide little discussion of circuit design and optimization. Similarly, Sinha et al. [93] extensively cover materials, fabrication technologies, and modeling methods of FET-based pH sensors, yet offer limited discussion on recent advancements in circuit design and their impact on performance. Baghini et al. [94] focus on ultra-thin ISFET sensing systems, discussing noise compensation circuits, flexible electronics, and readout circuit design, but lack specific coverage of the SiNW ISFET technology and a quantitative comparison of circuit-level optimization. In contrast, Moser et al. [95] explore recent trends in CMOS ISFET instrumentation, focusing on advancement in front-end circuit topologies for ISFET arrays and addressing key challenges such as offset and drift compensation. Nevertheless, their review does not include a detailed comparison of SiNW ISFET technology or recent improvements in readout circuit architectures tailored for this technology.

To address the limitations of previous reviews, this paper provides a targeted and in-depth analysis of SiNW ISFET readout circuits, particularly examining how circuit design choices impact key performance parameters such as sensitivity, noise immunity, linearity, and output range. Unlike prior works that primarily emphasize material properties and device fabrication, this study presents a quantitative and comparative evaluation of various readout circuit topologies, including conventional SiNW-based biosensors [8], sensitivity-enhanced structures [96], and SiNW/CMOS hybrid circuits [97]. The differences between this paper and previous reviews are summarized in Table 1. A detailed circuit-level analysis is conducted, focusing on current mirror-based circuits using P-type and N-type SiNW ISFETs [12,98], highlighting the trade-offs and performance improvements achieved through various design approaches. Additionally, this paper introduces a modified N-type readout circuit that integrates an operational amplifier (op-amp) with a negative feedback network to overcome the low sensitivity of conventional N-type designs. By optimizing the circuit topology, the proposed design enhances gain control, expands output range, and significantly reduces susceptibility to power supply noise while maintaining stability, making it more suitable for real-time and high-precision biosensor applications.

The organization of this paper is as follows. Section 2 reviews the key fabrication processes and methods of SiNW ISFETs. Section 3 explains the main properties of SiNW ISFETs as hydrogen ion sensors. Section 4 discusses key performance metrics and related theories of SiNW-based readout systems, along with an analysis of existing circuits. Section 5 introduces a modified N-type readout circuit and compares its performance with existing circuits to evaluate its potential. Finally, Section 6 summarizes the findings of this paper. For abbreviated terms used throughout, readers may refer to Table 2.

## 2. Fabrication Process of a SiNW ISFET

As a key component of semiconductor biochips, it is worth reviewing the major process steps in a SiNW ISFET. Various fabrication methods have been proposed and applied to implement a SiNW [8,17,47,63,99,100,101,102,103]. The SiNW ISFET, a pivotal component of semiconductor biochips, can be manufactured through two principal methodologies: the bottom-up and top-down. The bottom-up approach involves the synthesis of SiNWs from molecular precursors [104,105,106], allowing precise control of the dimensions and morphology of the nanowires at the angstrom to nanometer scale, as well as the production of intricate superlattice structures. On the other hand, this method faces significant challenges, including low device uniformity, poor manufacturing yield, and difficulties in CMOS integration, making it currently unsuitable for large-scale biosensor production.

In contrast, the top-down approach utilizes lithography-based techniques to shape SiNWs from bulk silicon wafers. This method is widely used due to its high manufacturing yield, cost-effectiveness, and seamless integration with the CMOS environment [48,61,107,108,109,110,111,112]. Particularly advantageous for large-scale production, this approach enables the fabrication of SiNW biosensors alongside CMOS readout circuits [8,48,100,101,102,103,113,114,115]. Table 3 provides a summarized comparison of the advantages and limitations of the bottom-up and top-down methods, including aspects not fully covered in the text.

**Figure 4:**
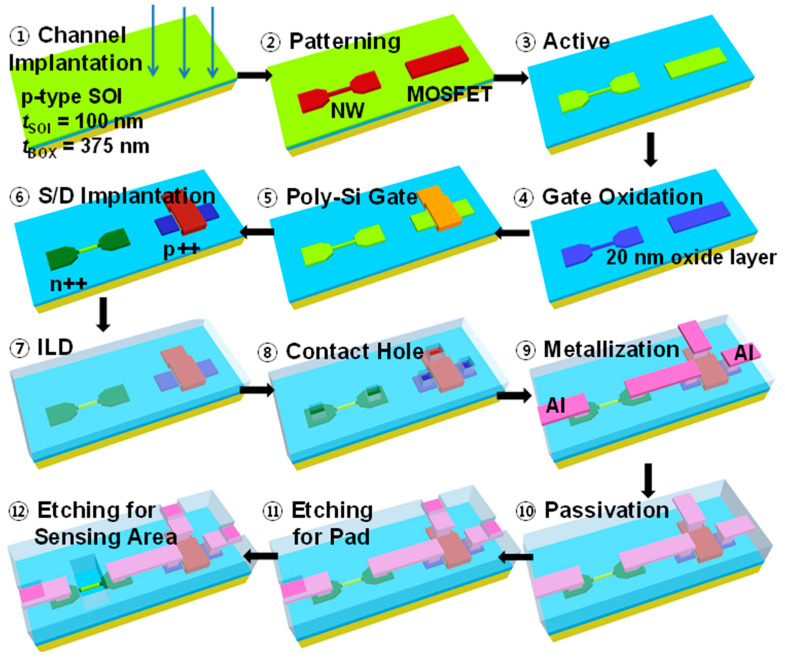
Major fabrication process flow of integrated SiNW ISFET and CMOS devices. Reprinted with CC BY 2.0 from Ref. [116]. 2014, Jieun Lee.

The top-down fabrication process of SiNW ISFETs integrated with CMOS devices is illustrated in Figure 4. The biosensors were fabricated on a boron-doped (4 × 10^15^ cm⁻^3^) 6-inch (100) silicon-on-insulator (SOI) wafer, with a 100 nm thick top Si layer separated from the Si substrate by a 375 nm thick buried oxide (BOX). The process begins with the growth of a 20 nm thick buffer oxide layer on the silicon surface for protection during channel implantation. Boron and phosphorus ions are implanted into the P-type and N-type regions, respectively, to define the channel structure (Step 1). After annealing, the active regions are patterned using a combination of e-beam lithography and photolithography (Step 2). Anisotropic etching is performed on the silicon layer with hydrogen silsesquioxane (HSQ) and photoresist (PR) masks, followed by inductively coupled plasma (ICP) etching in an HBr/O_2_ plasma, forming the SiNWs and CMOS regions (Step 3). Subsequently, the gate oxide layer is grown through dry oxidation at 850 °C, resulting in a 20 nm thick dielectric layer (Step 4). Poly-Si gates are deposited using low-pressure chemical vapor deposition (LPCVD) and patterned using photolithography (Step 5).

**Figure 5:**
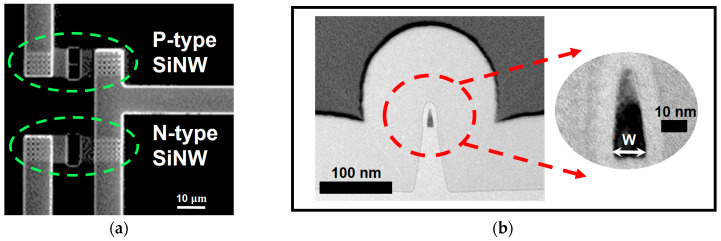
(**a**) Top view of N- and P-type SiNW ISFETs. (**b**) Cross-sectional view of the SiNW ISFET. Adapted with permission from Ref. [97]. 2013, Jieun Lee.

Then, the source and drain regions are doped with arsenic ions for N-type regions and boron ions for P-type regions via ion implantation, followed by high-temperature annealing for dopant activation (Step 6). A high-density plasma chemical vapor deposition (HDP-CVD) process is employed to deposit an inter-layer dielectric (ILD), which is planarized using chemical mechanical planarization (CMP) (Step 7). Contact holes are formed via photolithography and etching to expose the source, drain, and gate regions (Step 8). Metallization is performed through aluminum sputtering and patterning to create interconnections (Step 9), and a tetraethyl orthosilicate (TEOS) oxide passivation layer is deposited to protect the structures (Step 10). Finally, the sensing area of the SiNW channel is exposed by selectively etching the TEOS layer, enabling interaction with biomolecule analyzes, and the oxide on the metal pads is etched to expose the electrical contacts (Steps 11–12). Figure 5 presents a transmission electron microscopy (TEM) image of a cross-sectional view of the SiNW ISFET channel region [97].

## 3. SiNW ISFET as a Hydrogen Ion Sensor

**Figure 6:**
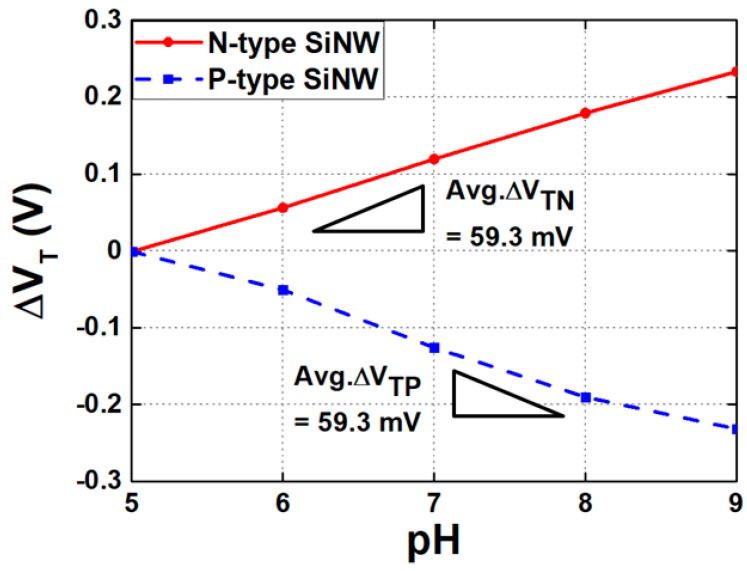
$V_{\mathrm{TH}}$ shift with respect to pH changes in N-type and P-type SiNW ISFETs.

The field effect that governs the operation of basic CMOSFETs also applies to SiNW biosensors. In an N-type ISFET, the source and drain regions are doped with N-type material, whereas the substrate consists of P-type material. As a complementary device, a P-type FET is fabricated using opposite-type dopants. The electrical properties of a SiNW ISFET are controlled by the gate voltage, which modulates channel conductance. For an N-type SiNW, the conductance of an ISFET decreases when the carrier is depleted by applying a low positive gate voltage, whereas it increases as the carrier is collected by applying a higher positive voltage. Due to the opposite polarity of carriers in N-type and P-type devices, the V_TH_ responds to pH concentration changes in reverse directions. Specifically, at lower pH levels (a smaller number of negatively charged ions), the gate of N-type SiNW collects more electrons in the channel region, lowering V_TH_. On the other hand, in a P-type SiNW, a lower pH level inhibits hole collection, increasing V_TH_. The electric field generated by ion binding at the gate functions similarly to an externally applied voltage. As illustrated in Figure 6, these characteristics enable SiNW ISFETs to function as electrical-based sensors, and they have been used as biosensors in various studies [92,117,118].

**Figure 7:**
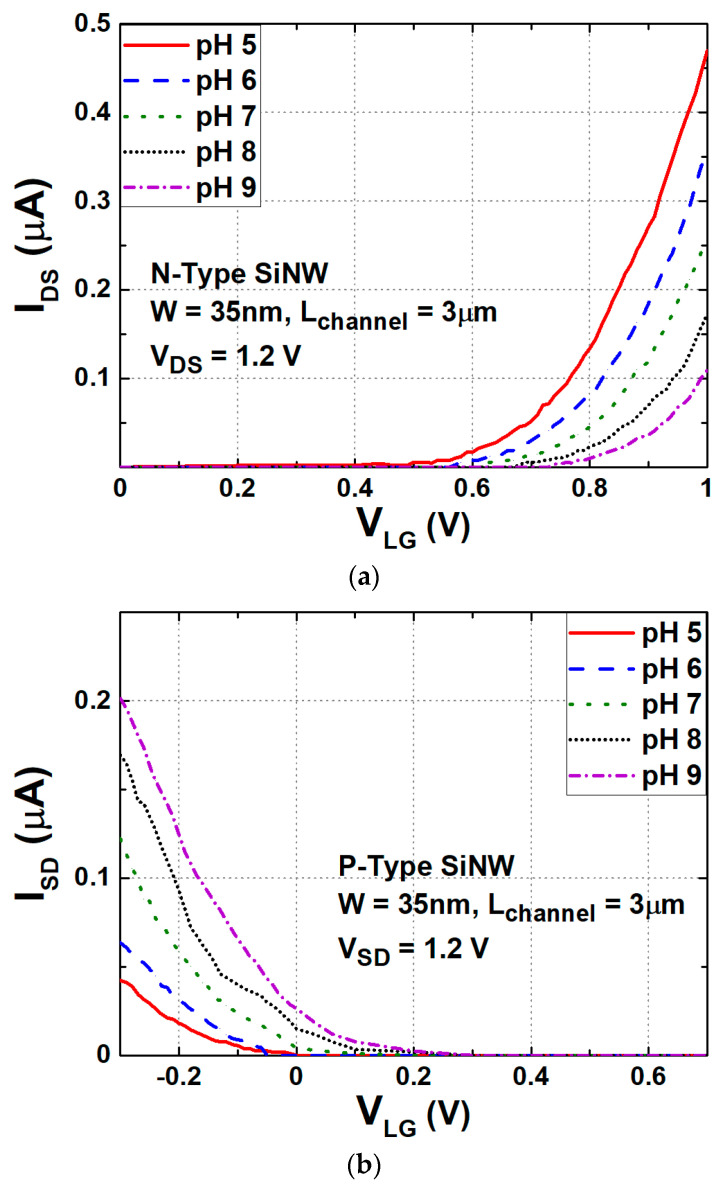
Transfer characteristics with respect to pH changes in (**a**) N-type and (**b**) P-type SiNW ISFETs.

A SiNW ISFET, a representative example of a FET-based biosensor, operates by controlling the conductance of the semiconductor channel through the gate voltage, thereby electrically connecting the source and drain. In pH concentration sensing, when the -NH2 group reaches the surface of the gate dielectric layer, it is either protonated to -NH3 or the surface is deprotonated to -SiO-, depending on the pH concentration. As a result, the interaction between the charged target molecule and the surface-immobilized receptor generates an electric field that influences the conductivity of the SiNW ISFET channel. When negatively charged target molecules bind to the receptors, positive carriers accumulate in the channel, whereas negative carriers accumulate when positively charged molecules bind. Therefore, as pH concentration increases, the V_TH_ of the N-type SiNW ISFET increases, whereas the V_TH_ of the P-type SiNW ISFET decreases, as shown in Figure 6. Since the drain currents (I_D_) are affected by V_TH_, similar to the conventional FETs, the transfer characteristics of a SiNW ISFET depend on pH concentration. Figure 7a,b illustrate the I_D_ of the N-type and P-type SiNW ISFETs versus gate voltages shown for different pH concentrations, respectively. Using these characteristics, many studies in the literature have investigated the design of readout circuits [97,116].

## 4. Circuit Design and Analysis of Readout Circuits

This section provides a comprehensive analysis of SiNW ISFET-based readout circuits, including conventional designs, SiNW/CMOS hybrid architectures, and current-mirror-based configurations. The discussion highlights their design methodologies, advantages, and key performance trade-offs to identify the most suitable approach for specific biosensor applications.

**Figure 8:**
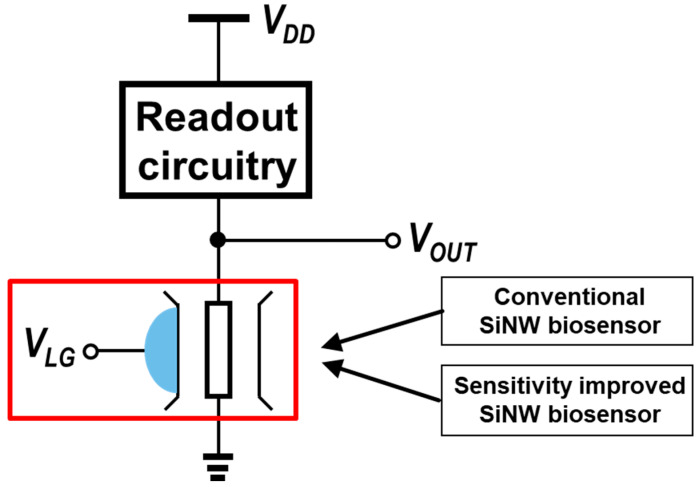
Conventional [8] and sensitivity-enhanced [96] SiNW ISFET readout circuits.

To utilize SiNW ISFETs as biosensors, various readout schemes have been proposed to detect changes in the V_TH_ of SiNW ISFETs under different pH concentrations (See Figure 8). Among them, the conventional SiNW ISFET circuit [8] employs a single-gate structure to detect pH changes by monitoring shifts in V_TH_ of the nanowire (Red box in Figure 8). Its design is simple and cost-effective, making it suitable for basic pH sensing applications. The improvement in sensitivity of this circuit is somewhat limited; however, small changes in V_TH_ result in relatively small differences in its current response.

The sensitivity-enhanced SiNW ISFET circuit [96] builds upon the conventional design by introducing a dual-gate configuration, which enhances electrostatic control over the nanowire and results in larger V_TH_ shifts (ΔV_TH1_ < ΔV_TH2_, where #1 refers to the conventional circuit and #2 refers to the sensitivity-enhanced SiNW ISFET circuit). Despite this improvement, the current sensitivity remains near the same (ΔI_1_ = ~ΔI_2_), and consequently, the output voltage sensing margin in the actual readout circuitry does not provide significant advantages (ΔV_OUT1_ = ~ΔV_OUT2_). Furthermore, the logic threshold voltage (V_LTH_), defined as the voltage level at which the logic state transitions, is determined by the V_TH_ shifts in N-type and P-type SiNW FETs. V_LTH_ is expressed as shown in Equation (1), which combines the V_TH_ shifts in both N-type and P-type transistors. These shifts, driven by pH-induced surface charge changes, make V_LTH_ a critical metric for assessing the stability and logic accuracy of SiNW-based circuits. Moreover, both circuits are susceptible to power supply noise, which may propagate during signal processing and compromise data acquisition reliability. These challenges underscore the importance of mitigating noise amplification and enhancing sensitivity in SiNW biosensor circuits.

(1) $$V_{\mathrm{LTH}}=\frac{V_{\mathrm{NTH}}+V_{\mathrm{PTH}}}{2}.$$

Building on these observations, a more advanced readout architecture is required to enhance sensing performance and improve noise robustness. Among various approaches, integrating SiNW ISFETs with CMOS technology presents a promising solution, combining the advantages of both platforms. To overcome the challenges of limited output voltage sensing margin and noise susceptibility, a hybrid biosensor circuit has been proposed [97]. By leveraging the strengths of both SiNW and CMOS devices, this approach enhances sensitivity while effectively suppressing noise, leading to improved overall sensor performance. The hybrid approach maximizes signal detection and minimizes noise, ensuring reliable biosensor operation.

**Figure 9:**
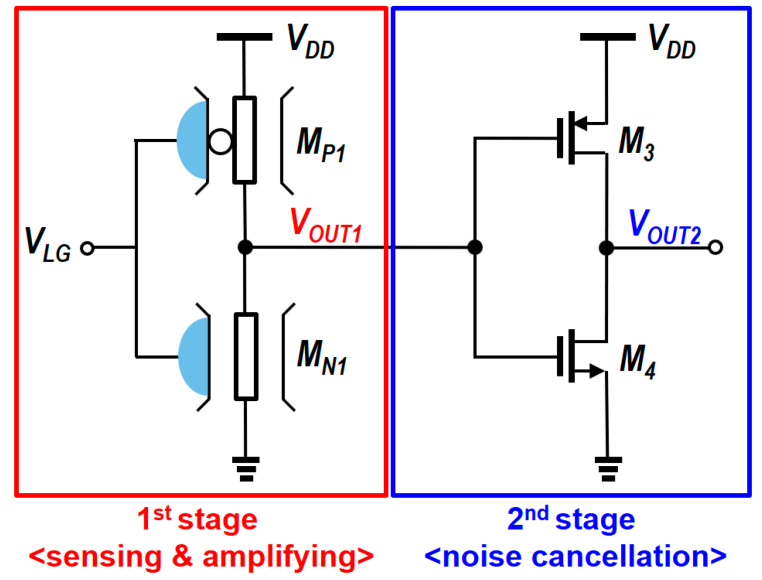
SiNW/CMOS hybrid biosensor for signal amplification and noise suppression. Adapted with permission from Ref. [97]. 2013, Jieun Lee.

**Figure 10:**
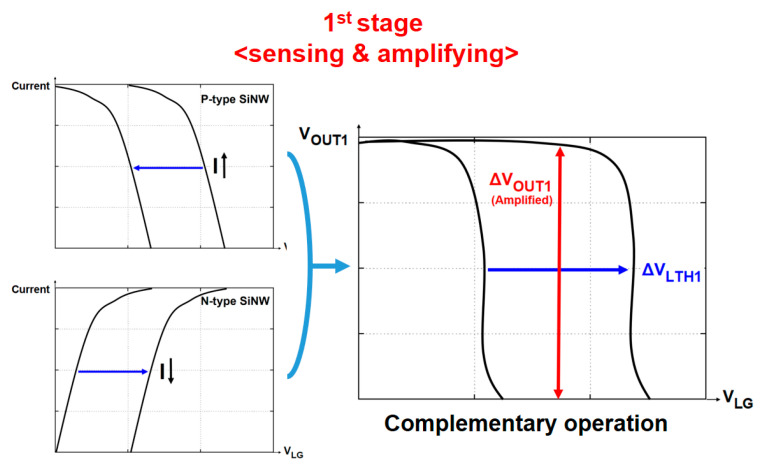
Complementary operation for signal amplification in the 1st stage. Adapted with permission from Ref. [97]. 2013, Jieun Lee.

The SiNW/CMOS hybrid circuit employs a two-stage architecture, as illustrated in Figure 9. In the first stage, the SiNW sensor detects and amplifies the initial signal by leveraging the complementary operation of N-type and P-type SiNWs to generate an amplified output voltage (ΔV_OUT1_), as shown in Figure 10. This complementary operation ensures that the sensing signals are reinforced, leveraging the voltage transfer characteristics of the SiNW sensor to achieve initial amplification. The second stage is designed to suppress supply noise by leveraging the opposite polarity in gain, further improving signal integrity.

The following section examines how this hybrid architecture achieves improved pH detection sensitivity and stabilizes the output by mitigating noise transfer. As pH concentration increases, the SiNW surface charge becomes more negative, inducing complementary V_TH_ shifts: a positive shift in the N-type SiNW FET and a negative shift in the P-type SiNW FET. These shifts collectively drive a positive change in the logic threshold voltage (V_LTH_). The measured V_LTH_ shift is approximately 50 mV per pH, closely matching the Nernst limit. This result confirms the effectiveness of the SiNW block in amplifying the output voltage (ΔV_OUT1_/ΔpH) in the first stage.

**Figure 11:**
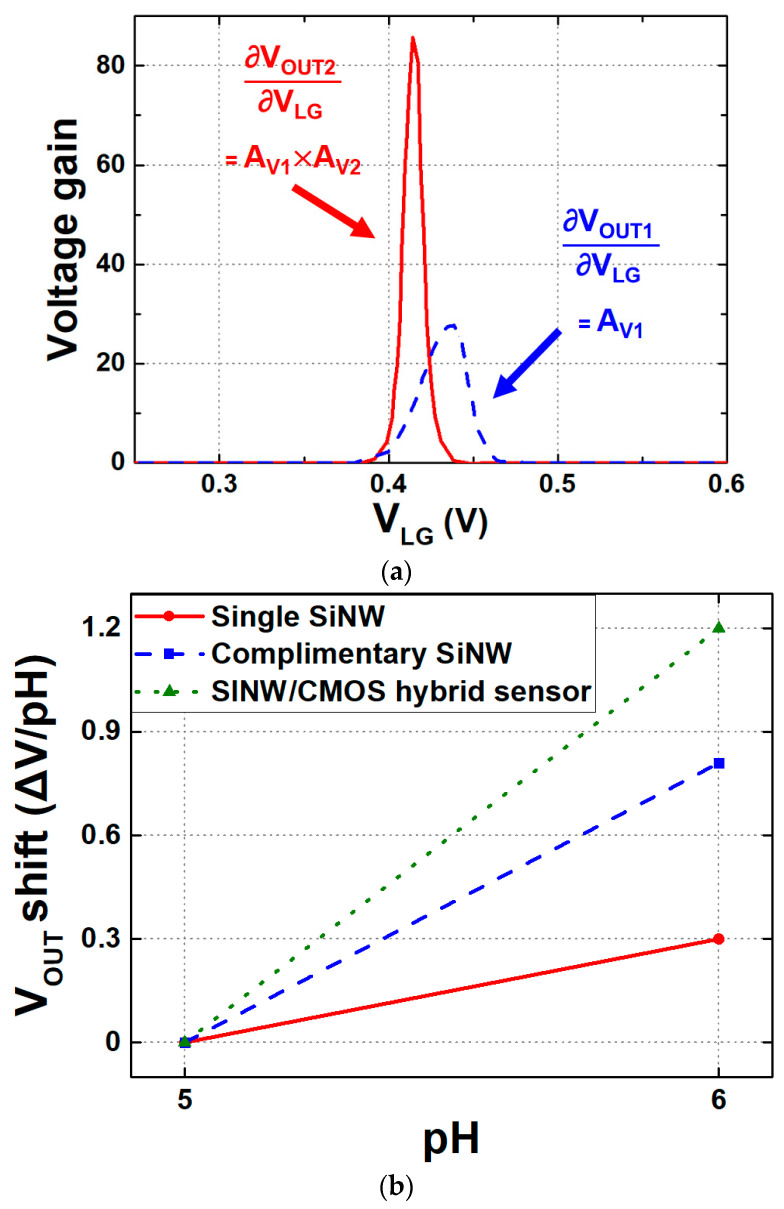
(**a**) Voltage gain characteristics of the SiNW/CMOS hybrid biosensor. (**b**) Output voltage sensitivity comparison of three biosensor types (V_DD_ = 1.2 V). Adapted with permission from Ref. [97]. 2013, Jieun Lee.

Figure 11a demonstrates how this shift translates into amplified output voltage sensitivity (ΔV_OUT1_/ΔpH) in the first stage. The complementary operation of N-type and P-type SiNW FETs not only shifts V_LTH_ but also enhances the output voltage per pH change beyond the Nernst limit. This is achieved through the voltage gain (A_V1_) provided by the SiNW block, which enhances the signal at this initial stage. The amplified output voltage shift in the first stage (ΔV_OUT1_/ΔpH) serves as the foundation for the second stage of amplification. As illustrated in Figure 11a, this initial amplification is critical for achieving the overall high sensitivity of the SiNW/CMOS hybrid biosensor, which is further enhanced in the CMOS stage. These results emphasize the critical role of the SiNW block in boosting sensitivity, enabling precise detection of even minor pH variations.

The amplification process in the SiNW/CMOS hybrid biosensor can be mathematically expressed as follows:

(2) $$\frac{\Delta V_{\mathrm{OUT}1}}{\Delta \mathrm{pH}}=\frac{\Delta V_{\mathrm{OUT}1}}{\Delta V_{\mathrm{LG}}}\cdot \frac{\Delta V_{\mathrm{LG}}}{\Delta V_{\mathrm{LT}}}\cdot \frac{\Delta V_{\mathrm{LT}}}{\Delta \mathrm{pH}}=A_{V1}\cdot 1\cdot \frac{\Delta V_{\mathrm{LTH}}}{\Delta \mathrm{pH}},$$

for the first stage, where A_V1_ represents the voltage gain of the SiNW block. In the second stage, the CMOS logic inverter further amplifies the signal as follows:

(3) $$\frac{\Delta V_{\mathrm{OUT}2}}{\Delta \mathrm{pH}}=\frac{\Delta V_{\mathrm{OUT}2}}{\Delta V_{\mathrm{OUT}1}}\cdot \frac{\Delta V_{\mathrm{OUT}1}}{\Delta \mathrm{pH}}=A_{V1}\cdot A_{V2}\cdot \frac{\Delta V_{\mathrm{LTH}}}{\Delta \mathrm{pH}}.$$

Combining both stages, the overall sensitivity of the hybrid biosensor can be expressed as follows:

(4) $$\Delta V_{\mathrm{OUT}2}=A_{V1}\cdot A_{V2}\cdot \Delta V_{\mathrm{OUT}1}.$$

This mathematical model highlights the synergistic amplification achieved through the combination of the SiNW and CMOS stages.

As illustrated in Figure 11b, the SiNW/CMOS hybrid biosensor exhibits a significant sensitivity improvement over conventional designs. This enhanced sensitivity results from the integration of the SiNW block and CMOS amplification stages, enabling the hybrid biosensor to achieve an output sensitivity of approximately 1.2 V/pH. This performance is markedly superior to that of the individual SiNW structure (0.3 V/pH) and the complementary SiNW block (0.81 V/pH). These results highlight the hybrid architecture’s ability to overcome the limitations of single-stage designs by leveraging dual-stage amplification for the detection of minor pH changes. Building on this enhanced architecture, it is anticipated that the circuit will be capable of generating highly sensitive output voltage signals even for minor pH variations.

To detect subtle biochemical changes, it is not only important to improve sensitivity but also to achieve a stable output while minimizing noise. In addition to delivering higher sensitivity, the hybrid SiNW/CMOS design increases the potential for robust noise suppression by using the CMOS inverter-type gain stage. Building on this concept, the following section explains how the hybrid biosensor effectively mitigates power supply fluctuations and maintains consistent performance under various operating conditions.

The SiNW/CMOS hybrid biosensor excels in noise reduction by integrating the CMOS inverter gain stage directly into the SiNW biosensor. With careful optimization of the second-stage gain, the average power supply noise can be suppressed, resulting in reduced voltage fluctuations. This design minimizes interference from the bulk electrolyte and the electrolyte–oxide interface, ensuring that noisy bio-signals are effectively filtered out. Experimental observations and TCAD simulations have validated the biosensor’s ability to produce clean, stable output signals in the presence of supply noise. This robustness across various environmental conditions underscores the reliability of the hybrid biosensor, establishing it as a practical platform for applications requiring precise biochemical sensing.

In addition to noise suppression and stability, another critical aspect of biosensor performance is the ability to provide accurate and reliable detection under varying conditions. These capabilities are key requirements for advanced applications such as genetic screening, early disease diagnostics, and personalized medicine. Thanks to its label-free detection method, there is no need for complex chemical labeling, which simplifies the sensing process while maintaining high accuracy. However, whereas the SiNW/CMOS hybrid biosensor significantly improves noise immunity, its ability to ensure precise detection across different environmental and process conditions remains a challenge. Variability in V_TH_ shifts and signal amplification can impact sensing performance, requiring further advances in circuit design to improve precision, linearity, and DR.

In order to address these challenges, advanced readout circuit architectures have been explored to enhance signal processing and detection accuracy. Among these, current-mirror-based circuits offer a promising solution for improving both performance and functionality in SiNW ISFET biosensors. By leveraging a reference pH level for comparison, this approach enables precise detection of target pH concentrations by analyzing differences in V_OUT_ caused by V_TH_ shifts. This method ensures that the output voltage reflects only the V_TH_ shifts, minimizing interference from other parameters such as mobility variations and background noise.

These circuits operate by comparing the reference pH level with the target pH concentration and detecting differences in output voltage (V_OUT_) as a result of V_TH_ shifts. By isolating the voltage response to V_TH_ variations, this method significantly reduces interference from external factors such as carrier mobility variations and background noise, leading to more reliable pH detection [98].

In this approach, the SiNW operates in the saturation region, where the I_D_ is governed by the MOSFET equation, as depicted in Equation (5). As V_TH_ changes with pH concentration, the corresponding variations in overdrive voltage result in proportional changes in I_D_, enabling precise pH sensing through current modulation. In this equation, μ_FE_ represents the mobility of the carriers in the SiNW, C_ox_ is the unit oxide capacitance, and V_G_ and V_S_ are the gate and source voltages of the SiNW. The current equation for a SiNW ISFET follows the standard MOSFET saturation region equation. Assuming the SiNW operates in the strong inversion region with negligible mobility degradation due to surface roughness, the current equation can be derived as follows:

(5) $$I_{\mathrm{SIN}W}=\frac{1}{2}\mu_{\mathrm{FE}}C_{\mathrm{ox}}\left(\frac{W_{\mathrm{eff}}}{L}\right){\left(V_{G}-V_{s}-V_{T.\mathrm{SIN}W}\right)}^{2}.$$

**Figure 12:**
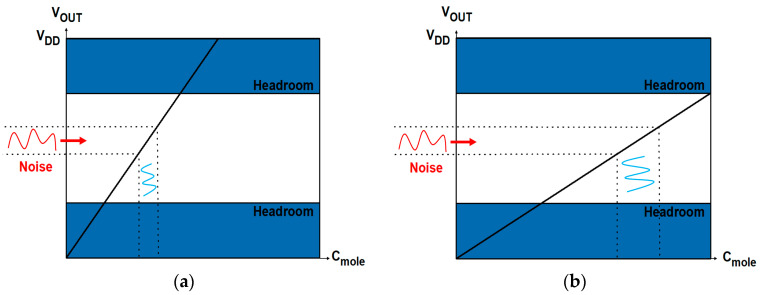
Impact of noise at the output on the mapped input ranges with (**a**) a high sensitivity and (**b**) a low sensitivity. Red and blue lines show input and output noise, respectively.

To evaluate the effectiveness of current-mirror-based readout circuits, key performance characteristics such as output range, sensitivity, linearity, and supply noise must be considered. The output range determines the maximum DR of the readout circuit. If the output range is too narrow, the DR may be limited, leading to distortion in the pH-dependent output. Even with a sufficiently wide output range, low sensitivity can be a limiting factor, making it difficult to detect low pH concentrations. For example, when interfacing with an analog-to-digital converter (ADC), insufficient sensitivity prevents setting a sufficiently small least-significant bit (LSB) for high-resolution operation. Additionally, lower sensitivity amplifies the perceived impact of output noise. As illustrated in Figure 12, a higher sensitivity readout circuit maps output noise to a narrower input range (Figure 12a), whereas lower sensitivity leads to a wider input range (Figure 12b) under the same output noise conditions. This observation highlights the critical trade-off between sensitivity and noise in biosensor design, emphasizing the need for careful noise management in high-precision applications.

For a comparative comparison and analysis, various performance metrics were evaluated using BSIM3 (Level-49) SiNW ISFET models and TSMC 180 nm standard CMOSFET models in a circuit simulator [119,120]. V_TH_ was extracted from the I_DS_ − V_LG_ characteristic curves at V_DS_ = 2 V. Both the N-type and P-type SiNW ISFETs were designed with the same W/L ratio of 200 nm/3 µm, and the CMOSFETs were also designed with the same W/L ratio of 1.5 µm/2 µm. For the operation of CMOS models in the readout circuits, the supply voltage (V_DD_) was set to 1.8 V [12].

**Figure 13:**
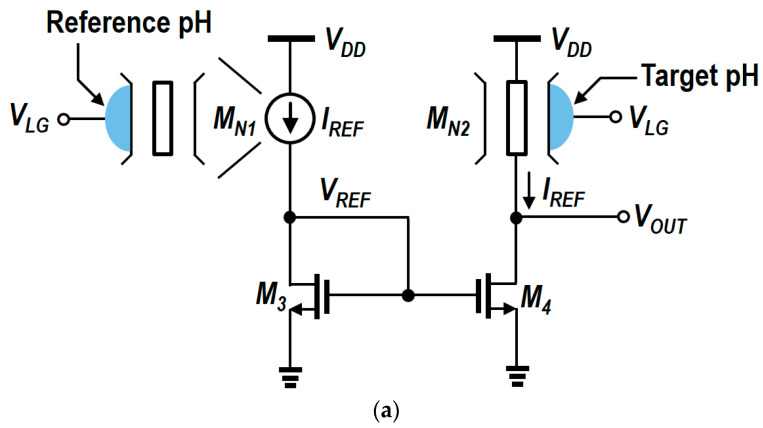

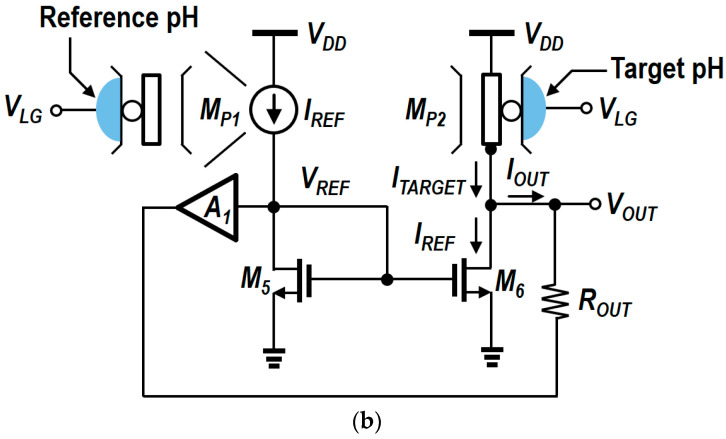
(**a**) Schematic of an N-type. Adapted with permission from Ref. [98]. 2016, Seungguk Kim. (**b**) Schematic of a P-type readout circuit. Adapted with permission from Ref. [12]. 2019, Sungju Choi.

Figure 13a,b illustrate the N-type and P-type readout circuits [12,98], respectively. The N-type readout circuit employs an N-type SiNW ISFET as a sensor and primarily uses a current-mirror configuration. The V_TH_ of the SiNW ISFET changes in response to variations in the input pH concentrations, which subsequently changes the V_OUT_. As all of the SiNW ISFETs and CMOSFETs in each readout circuit are designed and biased to operate in saturation, the output range is limited by the boundaries between the triode and saturation regions of each transistor. In the case of the N-type readout circuit (Figure 13a), the conditions for the saturation region of M_3_, M_4_, M_N1_, and M_N2_ are given by the following Equations (6) and (7). Equation (8) is the condition for enabling the operation of SiNW ISFETs.

(6) $$V_{\mathrm{REF}}-V_{\mathrm{TH},\mathrm{NMOS}}<V_{\mathrm{OUT}}.$$

(7) $$V_{\mathrm{LG}}-V_{\text{TH,N-SiNW}}<V_{\mathrm{DD}}.$$

(8) $$V_{\mathrm{LG}}-V_{\text{TH,N-SiNW}}>V_{\mathrm{OUT}}.$$

As shown in Equations (6)–(8), the output range of the N-type readout circuit is limited by the overdrive voltage of M_3_, M_4_, M_N1_, and M_N2_. Figure 13b illustrates the P-type readout circuit, which is based on a P-type SiNW ISFET and employs a current-mirror configuration similar to the N-type counterpart. The V_TH_ of the SiNW ISFET changes with pH variations, which determines the V_OUT_. Unlike the N-type circuit, a P-type SiNW ISFET is used as a sensor in the P-type readout circuit. The conditions under which NMOS transistors operate in saturation of the P-type readout circuit are identical to those in the N-type version. And the conditions under which a SiNW operates in the saturation region are shown in Equation (9).

(9) $${V_{\mathrm{DD}}-(V}_{\mathrm{LG}}+|V_{\text{TH,N-SiNW}}\left|\right)>V_{\mathrm{OUT}}.$$

**Figure 14:**
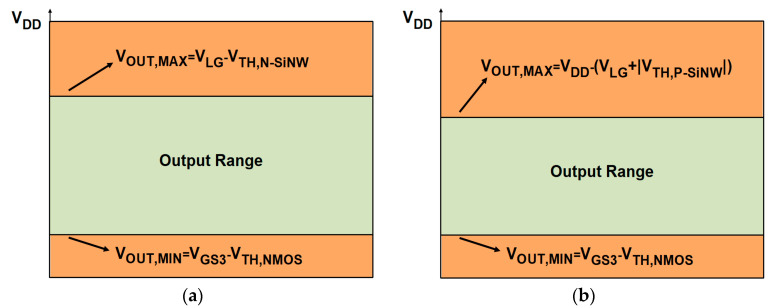
Output range of (**a**) N-type and (**b**) P-type readout circuits.

Consequently, the output range of the P-type readout circuits is constrained by the saturation conditions of NMOSFETs and SiNW ISFETs, as illustrated in Figure 14. In the simulation setup, the values of V_DD_, V_LG,P-SiNW_, V_TH,N-SiNW_, and V_TH,P-SiNW_ were 1.2, 0.35, 0.35, and 0.1 V, respectively. A comparison of the output ranges between the N-type and P-type ISFETs reveals that the output range of the P-type ISFET is comparatively smaller. This is mainly due to differences in V_TH_ and the resulting V_SAT_ levels. V_SAT_ in a MOSFET refers to the drain-source voltage (V_DS_) at which the device enters saturation, where I_D_ becomes stabilized and largely independent of V_DS_.

Even if the output range is large enough, the sensing performance is still significantly influenced by the sensitivity of the given readout scheme. A low sensitivity can lead to an increased influence of noise on the output node, thereby corrupting the quality of the output. Consequently, it is essential to analyze and optimize the sensitivity of the readout circuitry, as well as the output range and linearity. In the case of the N-type readout circuits, there is no amplification function as the M_2_ and M_4_ transistors act as source followers. Moreover, as M_4_ is not an ideal current source, it provides a voltage gain of less than unity. In [98], the sensitivity of the N-type readout circuit is approximately 50 mV/pH, which is similar to the variations in $V_{\mathrm{TH}}$ of the SiNW ISFET. In contrast, in the P-type readout circuit, SiNW ISFETs serve as a common-source amplifier with an NMOS current source as its load. Accordingly, the gain of the circuit can be modulated by the output resistance (R_OUT_). In [12], the sensitivity of the P-type readout circuit increases by approximately 90%, depending on the R_OUT_.

**Figure 15:**
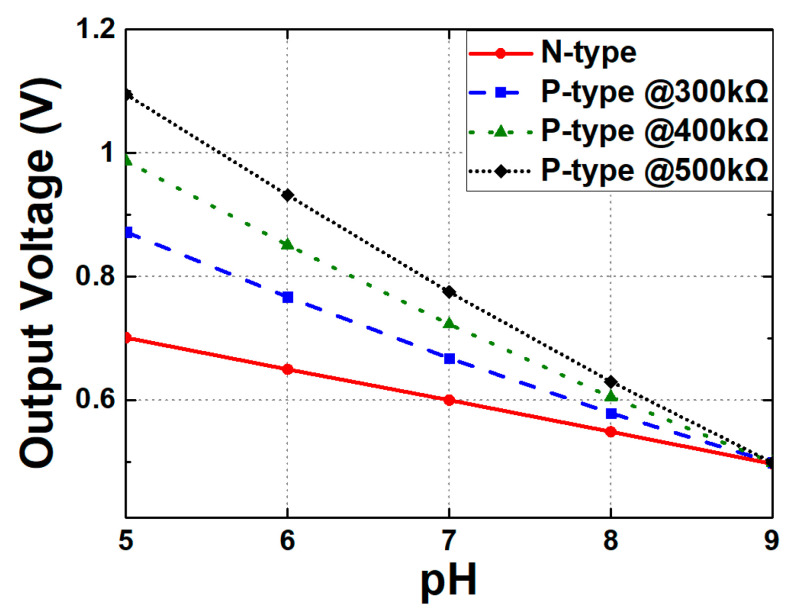
Output voltage changes according to the pH concentration of the N-type readout circuit and the P-type readout circuit when R_out_ is set to 300, 400, and 500 kΩ.

Figure 15 shows the V_OUT_ results for the N-type and P-type readout circuits. As illustrated, the N-type readout circuit exhibits a fixed and relatively low sensitivity due to the absence of an amplification stage. In contrast, the P-type readout circuit exhibits a significant improvement in sensitivity since the common-source stage provides signal gain. According to the simulations, adjusting the output resistance from 300 to 500 kΩ increases the sensitivity from 93 to a maximum of 149 mV/pH. In addition, the resistance can be optimized for the different input impedances of the subsequent stage. Regarding the selection of the optimal resistance of R_OUT_, the following aspects may be considered: First, increasing the R_OUT_ of the P-type readout circuit improves the sensitivity. In many cases, a higher sensitivity is preferred, but at the same time, a high sensitivity may put limitations on the range of V_OUT_, reducing the DR of readout circuits. Furthermore, increasing R_OUT_ potentially degrades the linearity characteristics of readout systems, which will be discussed in more detail in the following paragraph.

Linearity is an essential characteristic in readout circuits. Using an ADC after readout circuits can lead to increased quantization noise due to nonlinearity, thereby resulting in a decrease in SNR. Changes in V_OUT_ of the N-type readout circuit in response to the changes in V_TH_ of a SiNW due to pH variations can be calculated by Equations (10) and (11). Equation (11) shows that V_OUT_ is linear with respect to the V_TH_ of SiNW.

(10) $$\frac{B_{\mathrm{SiN}W}}{2}{\left(V_{\mathrm{LG}}-V_{\mathrm{REF}}-V_{\mathrm{TH},\mathrm{REF}}\right)}^{2}=\frac{B_{\mathrm{SiN}W}}{2}{\left(V_{\mathrm{LG}}-V_{\mathrm{OUT}}-V_{\mathrm{TH},\mathrm{TARGET}}\right)}^{2}.$$

(11) $$V_{\mathrm{OUT}}=V_{\mathrm{REF}}+V_{\mathrm{TH}}-V_{\mathrm{TH},\mathrm{TARGET}}=V_{\mathrm{REF}}+\Delta V_{\mathrm{TH}}.$$

As demonstrated in Equations (10) and (11), the final V_OUT_ is determined by pH variations detected by the sensor. The reference voltage (V_REF_) is a constant voltage with respect to V_TH_ of the MOSFET at the reference pH, serving as a baseline for pH measurement. The conduction parameter of a SiNW (B_SiNW_) is associated with mobility, gate capacitance, and device dimensions. The change in the V_OUT_ of the P-type readout circuit is expressed in Equation (13) and is not linear to V_TH_ of a SiNW.

(12) $$V_{\mathrm{OUT}}=V_{\mathrm{REF}}+I_{\mathrm{OUT}}R_{\mathrm{OUT}}=V_{\mathrm{REF}}+(I_{\mathrm{TARGET}}-I_{\mathrm{REF}})R_{\mathrm{OUT}}$$

(13) $$V_{\mathrm{OUT}}=V_{\mathrm{REF}}+R_{\mathrm{OUT}}\frac{B_{\mathrm{SiN}W}\;\cdot {\Delta V}_{\mathrm{TH}}}{2}(2(V_{\mathrm{DD}}-V_{\mathrm{LG}})-2|V_{\mathrm{TH}.\mathrm{REF}}|+\Delta V_{\mathrm{TH}})$$

In Equations (12) and (13), $I_{\mathrm{TARGET}}$ denotes the target current in the context of the circuit being described. This is defined as the current that flows through the SiNW ISFET when it is exposed to the target pH level or specific analyte concentration being measured. This current is used to determine the sensor’s response, as it reflects the changes in the sensor’s electrical properties due to the interaction with the target molecules or ions. The I_TARGET_ value is commonly compared to the reference current (I_REF_) to generate a corresponding output signal. The detailed derivation of Equation (13) is shown in Appendix A.

**Figure 16:**
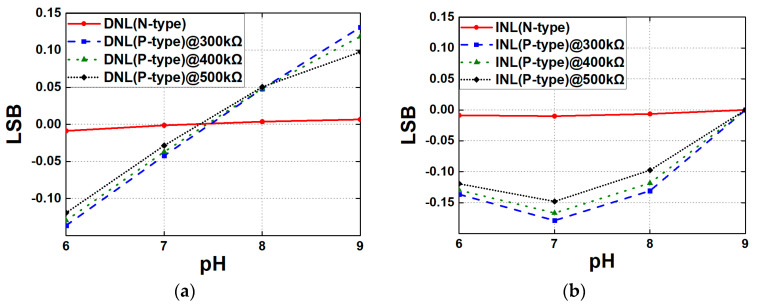
(**a**) DNL and (**b**) INL of the N-type and P-type readout circuits with different $R_{\mathrm{load}}$.

As demonstrated in Figure 16, the differential nonlinearity (DNL) and integral nonlinearity (INL) characteristics of the N-type and P-type readout circuits are exhibited under varying R_OUT_. Figure 16a illustrates that the N-type readout circuit demonstrates excellent linearity, exhibiting DNL values that are nearly equal to 0.009 LSB. In contrast, the P-type readout circuit shows considerably higher DNL values, reaching up to 0.13 LSB, suggesting a heightened risk of distortion or nonlinearity in the output signal. Figure 16b illustrates the INL values, which represent the cumulative deviations from ideal linearity across the entire output range. The N-type circuit demonstrates favorable INL performance, with values approximating 0.01 LSB, thereby indicating effective maintenance of overall linearity. In contrast, the P-type circuit exhibits considerably higher INL values, reaching up to 0.18 LSB and indicating a significant drawback in comparison with the N-type circuit. These findings imply that both circuits present their own advantages for pH-sensing applications. The N-type readout circuit, which exhibits superior linearity and minimal distortion, may be more suitable for applications where high accuracy and precision are required. On the other hand, the P-type circuit can provide enhanced sensitivity but may require additional calibration or tuning to offset its nonlinearity.

In addition to linearity considerations, the ability of a readout circuit to reduce power supply noise is a critical aspect of its performance, as this noise can significantly impact measurement accuracy. The current-mirror-based readout circuitry reviewed earlier generates single-ended outputs rather than differential signals, making it more susceptible to power supply fluctuations. In practical situations, a power supply includes unwanted fluctuations in addition to a DC voltage, and readout circuits may be sensitive to noise, which can degrade an SNR. Therefore, the supply noise dependence on V_OUT_ should be evaluated, and it can be calculated from a small-signal analysis. Equations (14) and (15) represent the changes in V_OUT_ resulting from supply voltage noise in the N-type and P-type readout circuits, respectively. By taking a partial derivative of V_OUT_ with respect to V_DD_, it is possible to obtain the following equations. These equations were derived using the small-signal models in Figure 13a,b, and the process is described in detail in Appendix B.

For the N-type,

(14) $$\frac{dV_{\mathrm{OUT}}}{dV_{\mathrm{DD}}}=\frac{1}{1+\frac{r_{o.\mathrm{SiN}W}}{r_{\mathrm{on}}}+g_{m.\mathrm{SiN}W}\;\cdot \;r_{o.\mathrm{SiN}W}}\approx \frac{1}{g_{m.\mathrm{SiN}W}\;\cdot r_{o.\mathrm{SiN}W}}.$$

For the P-type,

(15) $$\frac{{\mathrm{dV}}_{\mathrm{OUT}}}{{\mathrm{dV}}_{\mathrm{DD}}}=(\frac{g_{m.\mathrm{SiN}W}{\;\cdot \;r}_{o.\mathrm{SiN}W}+1}{r_{o.\mathrm{SiN}W}}{)(r}_{\mathrm{on}}{||r}_{o.\mathrm{SiN}W}{)\approx g}_{m.\mathrm{SiN}W}{\;\cdot \;(r}_{\mathrm{on}}{||r}_{o.\mathrm{SiN}W}).$$

In Equations (14) and (15), g_m.SiNW_ represents the transconductance of a SiNW ISFET, which denotes the rate of change in I_D_ with respect to the gate voltage. The parameter r_on_ indicates the on-resistance of the NMOS when the transistor is in the ON state. Meanwhile, r_o.SiNW_ represents the output resistance of a SiNW ISFET, which characterizes the resistance associated with the change in V_DS_ when I_D_ remains constant. These parameters have a considerable impact on the circuit’s sensitivity to power supply noise, as well as its overall linearity and performance.

**Figure 17:**
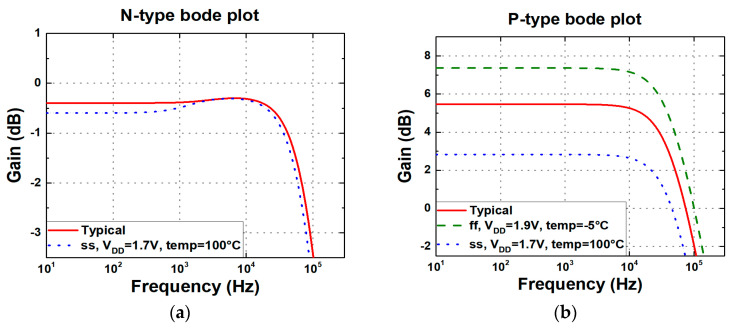
Bode plots of (**a**) N-type and (**b**) P-type readout circuits under different PVT conditions.

**Figure 18:**
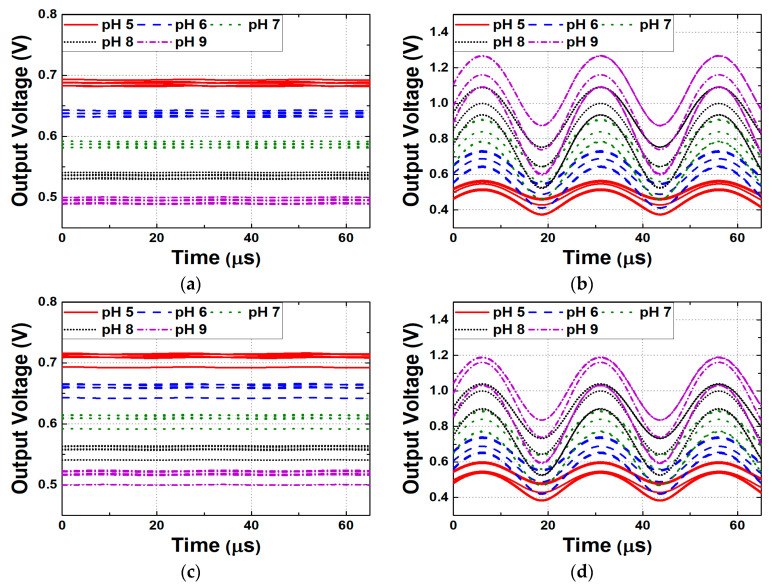
Output voltage variations due to the presence of supply noise in the (**a**) N-type readout circuit (temperature = −5 °C), (**b**) P-type readout circuit (temperature = −5 °C), (**c**) N-type readout circuit (temperature = 100 °C), (**d**) P-type readout circuit (temperature = 100 °C).

In the N-type readout circuits, the supply noise does not directly appear at the output as long as SiNW ISFETs are in the saturation region. Because the core structure is configured as a cascode stage, the impedance looking into the circuit from the power supply is boosted by the product of g_m_ of the SiNW ISFET and r_o_ of M_4_ in Figure 13a, reducing the impact of noise. Conversely, in P-type readout circuits, the power supply noise is amplified by the g_m.SiNW_ due to the common-source configuration. This noise is converted into a current and is multiplied by R_OUT_, resulting in the V_OUT_. Figure 17 and Figure 18, respectively, present the Bode plot of the N-type and P-type readout circuits and the output voltage waveform under power supply noise conditions. For the Bode plot simulation, a load capacitor of 10 pF was incorporated at the circuit output to ensure stability and to account for realistic loading conditions. Each simulation was conducted under PVT corner conditions, selecting both typical and worst-case scenarios. The PVT simulations encompassed a total of 27 cases, with transistor process corners (tt, ss, and ff), supply voltages (V_DD_ = 1.7 V, 1.8 V, and 1.9 V), and temperatures (−5 °C, 27 °C, and 100 °C) varied. In Figure 17, only the worst cases were selected for visualization. Figure 18 illustrates the worst-case scenarios—where the transistor operates in ss or ff process corners with V_DD_ = 1.7 V/1.9 V and temperature = −5 °C/100 °C—were highlighted. The power supply noise was modeled as a sinusoidal waveform with an amplitude of ±10% relative to V_DD_**.** In the N-type readout circuit, the supply voltage noise decreases to a 1% level. However, in the P-type readout circuit, the noise is amplified by more than twice, and the noise gain increases as R_OUT_ becomes larger. Therefore, as the sensitivity of the P-type readout circuit increases, its dependence on power supply noise also grows. Additionally, it is highly sensitive to PVT variations, and in worst-case scenarios, pH sensing fails to function properly.

The N-type and P-type readout circuits each present distinct trade-offs in terms of linearity, sensitivity, and noise resilience. While the N-type circuit ensures superior linearity (0.009 LSB/0.01 LSB) and noise immunity (1.2 mV variation), its sensitivity (~50 mV/pH) is limited due to the absence of an amplification stage. In contrast, the P-type circuit provides higher sensitivity (up to 149 mV/pH) but exhibits increased nonlinearity (0.13 LSB/0.18 LSB) and greater susceptibility to supply noise (343 mV variation). These trade-offs indicate that N-type circuits are more suitable for stability, while P-type circuits are better suited for applications requiring enhanced sensitivity.

## 5. Modified N-Type Readout Scheme

**Figure 19:**
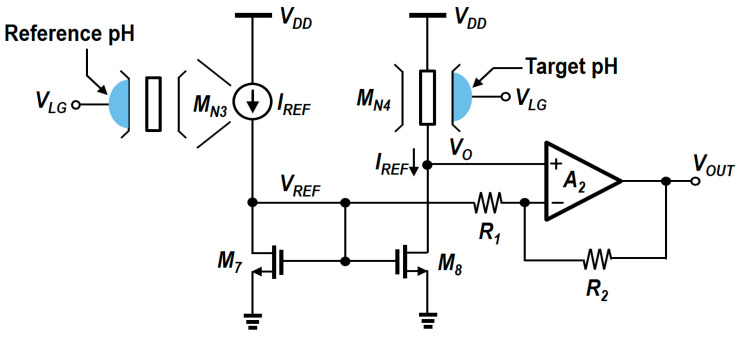
Schematic of the modified N-type readout circuit.

A comparative analysis and the results presented in the previous sections suggest that the N-type readout circuit exhibits good linearity and low dependence on power supply noise, but it is unable to control gain and sensitivity. On the other hand, the P-type readout circuit can adjust sensitivity but suffers from low linearity and high dependence on supply noise. To address the limitations of these readout circuits, the modified N-type readout circuit is proposed as a promising future direction in biosensor designs. As illustrated in Figure 19, the modified N-type readout circuit integrates an op-amp with a resistive feedback network (R_1_, R_2_). This integration enables precise control over sensitivity while maintaining the superior linearity and noise immunity of the conventional N-type design. This configuration enables precise adjustment of sensitivity through the ratio of R_1_ to R_2_, enhancing the circuit’s sensitivity without compromising linearity or introducing noise issues. In comparison to the P-type readout circuit, which allows for sensitivity adjustment but is highly susceptible to noise, this modified design retains the noise robustness of the N-type circuit while significantly enhancing detection capabilities.

This enhanced sensitivity is particularly advantageous in real-time biosensor applications, where the precise detection of small pH concentration changes is crucial. By effectively addressing the trade-offs between sensitivity, noise immunity, and linearity, the modified N-type readout circuit not only resolves the limitations of both N-type and P-type designs but also ensures high precision in pH detection. The modified circuit was designed using the same process as the N-type and P-type readout circuits discussed earlier.

(16) $$V_{\mathrm{OUT}}=\left(1+\frac{R_{2}}{R_{1}}\right)V_{O}-\frac{R_{2}}{R_{1}}V_{\mathrm{REF}}=V_{\mathrm{REF}}+\left(1+\frac{R_{2}}{R_{1}}\right)\cdot \;\Delta V_{\mathrm{TH}}.$$

(17) $$V_{O}=V_{\mathrm{REF}}+\Delta V_{\mathrm{TH}}.$$

**Figure 20:**
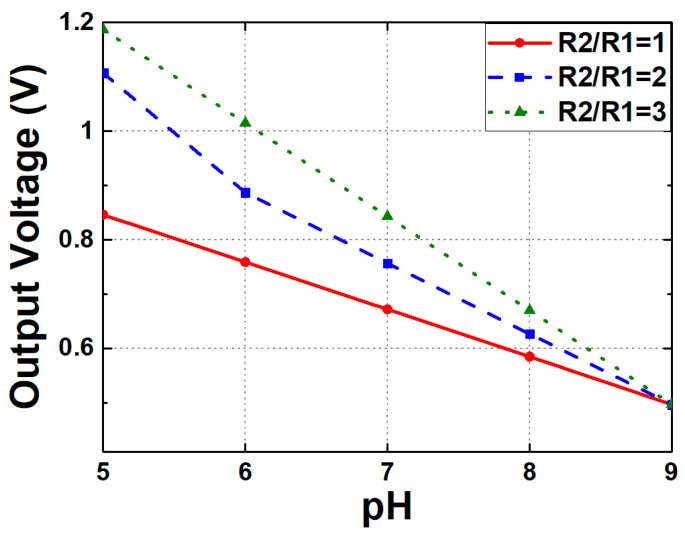
Simulated output voltages of the modified N-type readout circuit across varying pH concentrations and different R_2_/R_1_ ratios.

As indicated by Equations (16) and (17), the final V_OUT_ is determined by the pH variation detected by the sensor and is an amplified value produced by the operational amplifier A_2_. The intermediate output voltage (V_O_), which corresponds to the source voltage of M_N4_, represents the measured pH level and plays a crucial role in determining the final output voltage. The value of V_O_ is expressed as the sum of the reference voltage (V_REF_), which corresponds to a reference pH level, and the threshold voltage shift (ΔV_TH_) caused by the pH variation. ΔV_TH_ indicates the change in the threshold voltage at the target pH relative to the reference pH. V_REF_ is a fixed voltage related to the threshold voltage of the MOSFET at the reference pH and serves as a baseline for pH measurement. Figure 20 presents the simulation results of the change in output voltage with pH concentration when the ratio of R_1_ and R_2_ is 1, 2, or 3. The sensitivity of the proposed circuit was enhanced from 87 mV/pH to 172 mV/pH.

**Figure 21:**
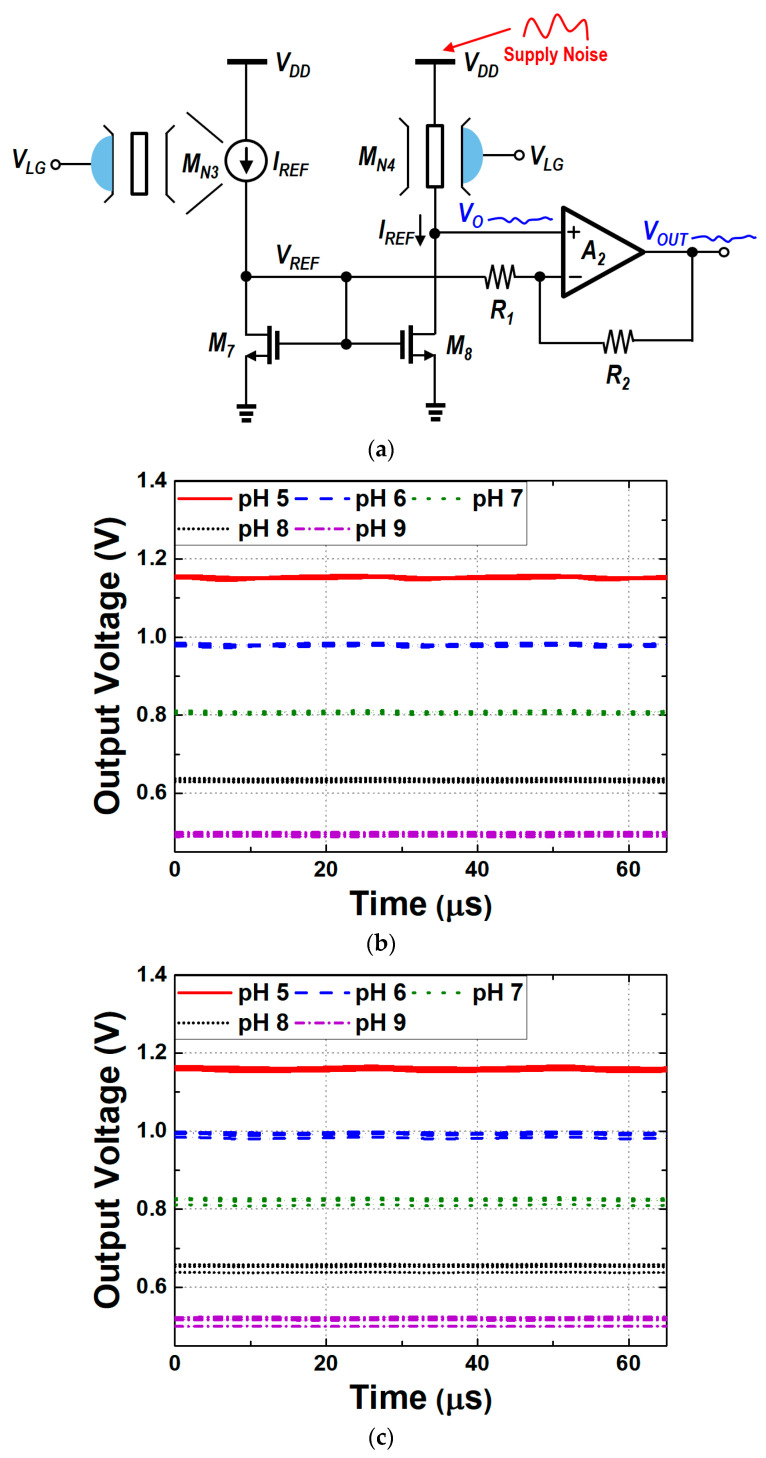
(**a**) Concept of supply noise immunity; (**b**) output voltage variations due to supply noise in the modified N-type readout circuits (temperature = −5 °C) (**c**) (temperature = 100 °C).

**Figure 22:**
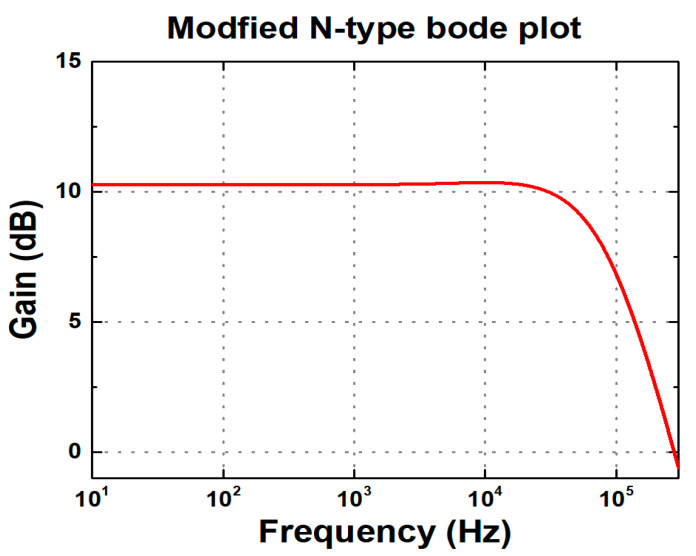
Bode plot of modified N-type readout circuits under different PVT conditions.

In scenarios where sensitivity is paramount, it is essential to regard noise as an external factor that can influence circuit performance. As previously discussed, the current-mirror type readout circuits do not utilize differential structures that facilitate supply noise cancelation. In the modified N-type readout, the circuit may exhibit sensitivity to supply noise, which can cause fluctuations in the output voltage. Therefore, the supply noise dependence of the V_OUT_ should be carefully examined through the implementation of appropriate small-signal analysis. Additionally, as there is no DC current flowing into the op-amp, the impact of supply noise on the output can be described by Equation (14). Since the modified N-type readout circuit adopts the structure of the existing N-type design, Equation (13) may also be applied. As mentioned earlier, the cascode structure of the N-type readout circuit provides inherent shielding against supply noise, ensuring that the output voltage remains largely insensitive to such variations. Figure 21a illustrates the concept of supply noise immunity in the modified N-type readout circuit. The cascode configuration effectively shields the circuit from power supply noise, reducing its impact on the output voltage. Figure 21b,c present the PVT corner simulation output voltage waveforms under power supply noise conditions at temperatures of −5 °C and 100 °C, respectively. Under the same percentage V_DD_ variation as in Figure 18, the peak-to-peak output voltage variations for the modified N-type readout circuit were observed to be about 5 mV, which is comparable to the peak-to-peak output voltage variations seen in the current-mirror-based N-type readout circuit. Figure 22 presents the Bode plot of the modified circuit. The PVT simulation results indicate that the variations in gain and bandwidth remained within 2% compared to the typical case, even under worst-case conditions; therefore, they were not included in the figure. Finally, Table 4 summarizes the key differences between the two current mirror-based readout circuits and the modified readout circuit.

The modified readout circuit demonstrates high linearity and excellent noise immunity, making it a promising design for various biosensor applications. However, design constraints, such as the accuracy of resistors and the offset voltage of op-amps, may potentially affect resolution. These limitations could be addressed through alternative configurations, such as replacing resistors with switched-capacitor structures, which are expected to further enhance the circuit’s performance. With these improvements, the proposed circuit could offer a more robust and reliable solution for precise biochemical sensing across diverse applications.

## 6. Conclusions

This paper presents a comprehensive review of SiNW ISFET-based readout circuits designed for biosensor applications. First, the fabrication methods and unique characteristics of SiNW ISFETs are explained, followed by a discussion of the performance metrics associated with different types of readout circuits. The performance parameters, including dynamic (output) range, sensitivity (gain), linearity, and supply noise immunity, are investigated for the conventional types of readout circuits. In addition, the properties and features of SiNW/CMOS hybrid and current-mirror-based readout circuits are investigated with a detailed analysis.

Based on these findings, the notion of a modified N-type readout circuit is introduced as a prospective future direction. By addressing key limitations in existing designs, this approach underscores the potential for optimizing biosensor performance. Further research is essential to validate the expected advantages, refine its implementation, and ensure its effectiveness in a variety of practical applications. These efforts will lay the foundation for the development of more reliable and versatile biosensor technologies.

## Figures and Tables

**Table 1:** Comparison of existing review papers and this study.

Paper	Main Research Focus	Limitations and Differences from This Study
Cao et al. [92]	ISFET sensors for biomolecule detection and commercialization	Existing reviews focus on ISFET applications, materials, and fabrication but lack a detailed circuit-level analysis, including design strategies, optimization techniques, and their impact on sensitivity, noise immunity, and linearity.
Sinha et al. [93]	Materials, fabrication, and modeling methods for FET-based pH sensors	
Baghini et al. [94]	Ultra-thin ISFET sensor systems, noise compensation, and flexible electronics	
Moser et al. [95]	CMOS ISFET instrumentation and front-end circuit design	
This Study	SiNW ISFET readout circuit analysis, performance evaluation, and proposal of a novel N-type circuit	Provides a comprehensive circuit-level analysis and optimization. Proposes a novel N-type readout circuit to enhance sensitivity while maintaining stability.

**Table 2:** List of abbreviated words.

Abbreviation	Definition
ADC	Analog-to-Digital Converter
BOX	Buried Oxide
B_SiNW_	Conduction Parameter of SiNW
CMOS	Complementary Metal Oxide Semiconductor
CMP	Chemical Mechanical Planarization
C_mole_	Concentration Range of Target Biomolecules
C_ox_	Unit Oxide Capacitance
DNL	Differential Nonlinearity
DR	Dynamic Range
FET	Field-Effect Transistor
g_m,SiNW_	Transconductance of the SiNW ISFET
GFET	Graphene Field-Effect Transistor
HDP-CVD	High-Density Plasma Chemical Vapor Deposition
HSQ	Hydrogen Silsesquioxane
ICP	Inductively Coupled Plasma
I_D_	Drain Current
ILD	Inter-Layer Dielectric
INL	Integral Nonlinearity
I_REF_	Reference Current
ISFET	Ion-Sensitive Field-Effect Transistor
LPCVD	Low-Pressure Chemical Vapor Deposition
LSB	Least-Significant Bit
Op-Amp	Operational Amplifier
PR	Photoresist
PVT	Process, Voltage, and Temperature
R_OUT_	Output Resistance
SiNW	Silicon Nanowire
SNR	Signal-to-Noise Ratio
SOI	Silicon-on-Insulator
TEM	Transmission Electron Microscopy
TEOS	Tetraethyl Orthosilicate
V_DD_	Supply Voltage
V_DS_	Drain-Source Voltage
V_LTH_	Logic Threshold Voltage
V_OUT_	Output Voltage
V_REF_	Reference Voltage
V_TH_	Threshold Voltage
$\mu_{\mathrm{FE}}$	Mobility of Carriers
r_o,SiNW_	Output Resistance of the SiNW ISFET

**Table 3:** Comparison of top-down and bottom-up method of SiNWs.

	Top-Down Method	Bottom-Up Method
Fabrication Process	SiNWs are synthesized from molecular precursors rather than bulk semiconductor wafers, enabling the fabrication of complex superlattice structures	SiNWs are produced from molecular precursors by using a metal nano-cluster mediated vapor-liquid-solid mechanism
Advantages	Characterized by high yield and optimized for large-scale production	Enables flexibility in selecting materials for nanowire synthesis
	Provides reliable and consistent synthesis processes	Facilitates in situ doping with diverse dopants for tailored electronic properties
	Seamlessly integrates with CMOS technology and other systems	Supports the synthesis of SiNWs with diameters smaller than 10 nm
Limitations	Requires extensive processing time	Achieving uniformity in SiNWs bridging source and drain electrodes is highly challenging.
	Significant silicon waste during etching	Device assembly needs precise pre-alignment and placement of SiNWs, causing integration challenges
	Challenges in achieving sub-10 nm structures with lithography	Mass production of SiNW devices remains impractical

**Table 4:** Comparison table of current-mirror-based readout circuits and modified readout circuit.

	N-Type	P-Type	Improved N-Type
Number of circuit components	2 × NMOS/2 × SiNW	2 × NMOS/2 × SiNW/ 1 × Buffer/1 × R	2 × NMOS/2 × SiNW/ 1 × op-amp/2 × R
Linearity (DNL/INL)	0.009 LSB/0.01 LSB	0.13 LSB/0.18 LSB	0.003 LSB/0.003 LSB
Sensitivity increase rate, max value (%, mV/pH)	0, 51	60, 149 @ 500 kΩ	98, 172 @ ratio = 3
Output voltage range	V_DD_ − (V_LG_ − V_TH,N-SiNW_ + V_OV_^1^_,NMOS_)	V_DD_ − (V_OV,P-SiNW_ + V_OV,NMOS_)	V_DD_ − (V_LG_ − V_TH,N-SiNW_ + V_OV,NMOS_)
Linearity (DNL/INL)	0.009 LSB/0.01 LSB	0.13 LSB/0.18 LSB	0.003 LSB/0.003 LSB
Supply induced output variations (±10% on top of V_DD_, mV)	1.2	343	5.1
Gain (dB)	−0.4	5.5	10.3
3 dB bandwidth (kHz)	112.2	46.3	92.5
Power consumption (μW)	4.13	4.32 + 1.8 (op-amp)	4.30 + 1.8 (op-amp)

^1^ V_OV_ = V_GS_ − V_TH_.

**Figure A1:**
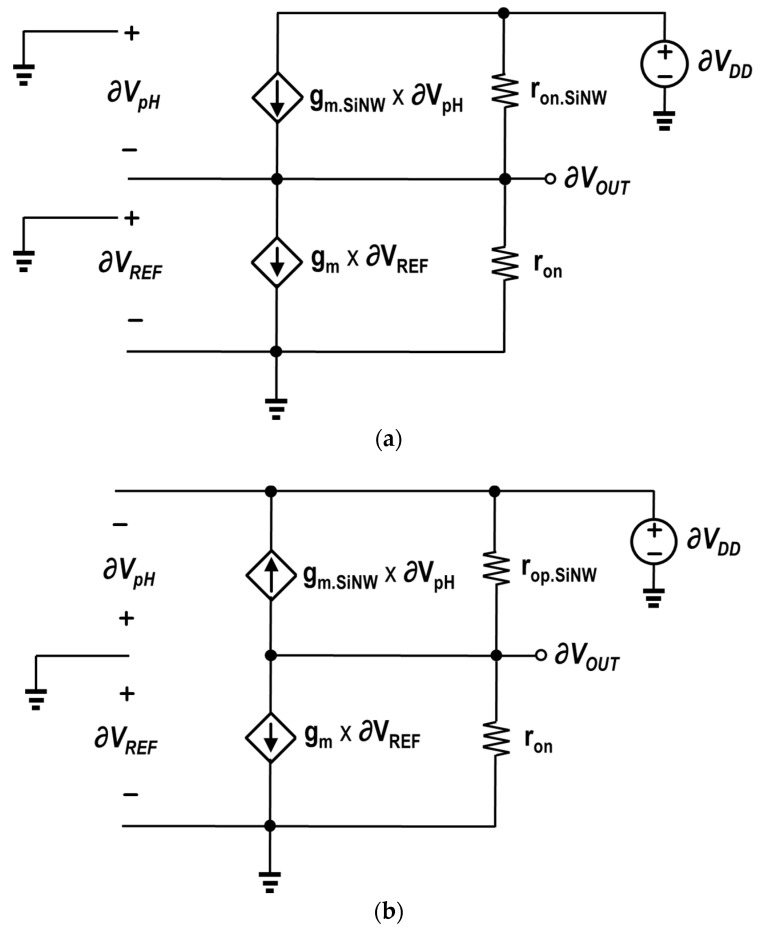
Small-signal model in (**a**) N-type readout circuit and (**b**) P-type readout circuit.

## Data Availability

No new data were created or analyzed in this study.

## Appendix A

This section provides a detailed development of Equation (13) used in Section 4. The purpose of this appendix is to provide an explanation of the thesis to better understand these equations for readers.

First, let I_REF_ be the current when the PMOS has a reference threshold voltage (V_TH,REF_) and I_REF_ can be expressed as in Equation (A1). In the reference situation, V_SG_ = V_DD_ − V_LG_ and V_TH_ = V_TH,REF_. Therefore, I_REF_ can be rewritten as Equation (A2), and for convenience, letting V_OV_ = (V_DD_ − V_LG_) − |V_TH,REF_|, I_REF_ is simplified as Equation (A3).

(A1) $$I_{\mathrm{REF}}=\frac{B_{\mathrm{SiN}W}}{2}{\left(V_{\mathrm{SG}}-{|V}_{\mathrm{TH}}|\right)}^{2}.$$

(A2) $$I_{\mathrm{REF}}=\frac{B_{\mathrm{SiN}W}}{2}{\left(V_{\mathrm{DD}}-{V_{\mathrm{LG}}-|V}_{\mathrm{TH},\mathrm{REF}}|\right)}^{2}.$$

(A3) $$I_{\mathrm{REF}}=\frac{B_{\mathrm{SiN}W}}{2}({V_{\mathrm{OV}})}^{2}.$$

If we assume that the V_TH_ has shifted by ΔV_TH_ due to a change in pH, the effective threshold voltage will be |V_TH,REF_| − ΔV_TH_. The result is V_SG_ − V_TH_, as shown in Equation (A4). This can be represented as Equation (A5), which when expanded becomes Equation (A6).

(A4) $${\left(V_{\mathrm{SG}}-{|V}_{\mathrm{TH}}|\right)}_{\mathrm{SENSOR}}=\{(V_{\mathrm{DD}}-V_{\mathrm{LG}})-(|V_{\mathrm{TH},\mathrm{REF}}|-{∆V}_{\mathrm{TH}})\}.$$

(A5) $$I_{\mathrm{TARGET}}=\frac{B_{\mathrm{SiN}W}}{2}{\left(V_{\mathrm{OV}}-{∆V}_{\mathrm{TH}}\right)}^{2}.$$

(A6) $$I_{\mathrm{TARGET}}=\frac{B_{\mathrm{SiN}W}}{2}\left\{{V_{\mathrm{OV}}}^{2}+{2V_{\mathrm{OV}}∆V}_{\mathrm{TH}}+{\left({∆V}_{\mathrm{TH}}\right)}^{2}\right\}.$$

If the sensor produces an output voltage by the difference between I_TARGET_ and I_REF_, then I_TARGET_-I_REF_ can be represented as Equations (A7) and (A8).

(A7) $$I_{\mathrm{TARGET}}-I_{\mathrm{REF}}=\frac{B_{\mathrm{SiN}W}}{2}\left\{{V_{\mathrm{OV}}}^{2}+{2V_{\mathrm{OV}}∆V}_{\mathrm{TH}}+{\left({∆V}_{\mathrm{TH}}\right)}^{2}\right\}-\frac{B_{\mathrm{SiN}W}}{2}{\left(V_{\mathrm{OV}}\right)}^{2}.$$

(A8) $$I_{\mathrm{TARGET}}-I_{\mathrm{REF}}=B_{\mathrm{SiN}W}{V_{\mathrm{OV}}∆V}_{\mathrm{TH}}+\frac{B_{\mathrm{SiN}W}}{2}{{(∆V}_{\mathrm{TH}})}^{2}.$$

If the schematic assumes that the output node flows current (I_TARGET_ − I_REF_) through R_OUT_ for V_REF_, then Equation (A9) holds. Substituting Equation (A8) into Equation (A9) at this stage yields Equation (A10), which is consistent with Equation (13).

(A9) $$V_{\mathrm{OUT}}=V_{\mathrm{REF}}+I_{\mathrm{OUT}}R_{\mathrm{OUT}}=V_{\mathrm{REF}}+(I_{\mathrm{TARGET}}-I_{\mathrm{REF}})R_{\mathrm{OUT}}.$$

(A10) $$V_{\mathrm{OUT}}=V_{\mathrm{REF}}+R_{\mathrm{OUT}}\frac{B_{\mathrm{SiN}W}\;\cdot {\Delta V}_{\mathrm{TH}}}{2}(2(V_{\mathrm{DD}}-V_{\mathrm{LG}})-2|V_{\mathrm{TH}.\mathrm{REF}}|\;+\Delta V_{\mathrm{TH}}).$$

## Appendix B

This section provides a detailed development of Equations (14) and (15) used in Section 4. The purpose of this appendix is to facilitate the understanding of these equations, and we have presented the development in as much detail as possible.

First, the small-signal model of the circuit in Figure 13a to arrive at Equation (14). In the circuit in Figure 13a, the small-signal model for the output stage is shown in Figure A1a. From Figure A1a, the expression for the current flowing from V_DD_ to V_OUT_ can be written as Equation (A11), which can be simplified to Equation (A12). This is consistent with Equation (14).

(A11) $$\frac{dV_{\mathrm{OUT}}}{r_{\mathrm{on}}}=g_{m.\mathrm{SiN}W}\;\cdot \;dV_{\mathrm{OUT}}+\frac{dV_{\mathrm{DD}}-dV_{\mathrm{OUT}}}{r_{\mathrm{on},\mathrm{SiN}W}}.$$

(A12) $$\frac{dV_{\mathrm{OUT}}}{dV_{\mathrm{DD}}}=\frac{1}{1+\frac{r_{on.\mathrm{SiN}W}}{r_{\mathrm{on}}}+g_{m.\mathrm{SiN}W}\;\cdot \;r_{on.\mathrm{SiN}W}}\approx \frac{1}{g_{m.\mathrm{SiN}W}\;\cdot r_{on.\mathrm{SiN}W}}.$$

Figure 13b can be analyzed in a similar way. Figure A1b shows the small-signal model of the output stage in Figure 13b, and as before, an expression for the current flowing from V_DD_ to V_OUT_ can be formulated as Equation (A13). This can be simplified to look like Equation (A14), which is consistent with Equation (15).

(A13) $$\frac{dV_{\mathrm{OUT}}}{r_{\mathrm{on}}}=g_{m.\mathrm{SiN}W}\;\cdot \;dV_{\mathrm{DD}}+\frac{dV_{\mathrm{DD}}-dV_{\mathrm{OUT}}}{r_{\mathrm{op}.\mathrm{SiN}W}}.$$

(A14) $$\frac{{\mathrm{dV}}_{\mathrm{OUT}}}{{\mathrm{dV}}_{\mathrm{DD}}}=(\frac{g_{m.\mathrm{SiN}W}\;{\cdot \;r}_{op.\mathrm{SiN}W}+1}{r_{op.\mathrm{SiN}W}})(r_{\mathrm{on}}\left|\right|r_{\mathrm{op}.\mathrm{SiN}W})\;\approx \;g_{m.\mathrm{SiN}W}\cdot (r_{\mathrm{on}}{||r}_{op.\mathrm{SiN}W}).$$
